# The Role of High-Sensitivity Cardiac Troponin and Ischemia-Modified Albumin in Patients with Lower Extremity Peripheral Arterial Disease

**DOI:** 10.3390/ijms262211214

**Published:** 2025-11-20

**Authors:** Vinko Boc, Aleš Blinc, Anja Boc

**Affiliations:** 1 Department of Vascular Diseases, University Medical Centre Ljubljana, 1000 Ljubljana, Slovenia; vinko.boc@kclj.si (V.B.); ales.blinc@kclj.si (A.B.); 2 Department of Internal Medicine, Faculty of Medicine, University of Ljubljana, 1000 Ljubljana, Slovenia; 3 Institute of Anatomy, Faculty of Medicine, University of Ljubljana, 1000 Ljubljana, Slovenia

**Keywords:** peripheral arterial disease, biomarkers, high-sensitivity troponin, ischemia-modified albumin, risk stratification

## Abstract

Peripheral arterial disease (PAD) is a growing global health concern associated with substantial morbidity, mortality, and healthcare costs. Prognostic stratification is currently based largely on clinical presentation, but patients with similar symptoms can have heterogeneous outcomes. Reliable biomarkers could improve the risk assessment of PAD patients and enable individualized patient management. High-sensitivity cardiac troponins (hs-cTn) and ischemia-modified albumin (IMA) have emerged as promising candidates. Hs-cTn appears to correlate with PAD severity and predict major adverse limb and cardiovascular events, likely reflecting subclinical myocardial injury in this patient population. Less extensively studied, IMA reflects oxidative stress, acidosis, and free radical activity. Its levels also seem to correlate with PAD severity, increasing with more advanced PAD. Notably, patients with advanced PAD but undetectable levels of biomarkers might have prognoses similar to those with milder disease, suggesting potential incremental prognostic value over clinical assessment. Despite these associations, the current applicability of hs-cTn and IMA remains limited by heterogeneous cut-off definitions and the absence of randomized controlled trials in the PAD population. Standardizing biomarker thresholds and prospective validation are crucial before integrating them into clinical practice. Both hs-cTn and IMA hold promise as tools for refined risk stratification in PAD, warranting further research.

## 1. Introduction

Lower extremity peripheral arterial disease (PAD) affects over 230 million people worldwide and represents the third most common clinical manifestation of advanced atherosclerosis, following coronary artery disease and ischemic stroke [1]. Despite increasing public awareness of the consequences and risk factors for atherosclerosis, the global number of individuals with PAD almost doubled between 1990 and 2019, paralleled by an almost two-fold increase in PAD-associated mortality [2].

As atherosclerosis is a chronic, progressive disease, with plaque formation beginning as early as childhood and developing over the years [3], the likelihood of its clinical manifestations increases with age. In 2019, the global prevalence of PAD was 1.5% among individuals aged ≥40 years, rising to 14.9% in those aged 80–84 years [4]. Since atherosclerosis is a systemic disease, plaques can also be present in other arterial beds. Polyvascular disease is, in fact, the most common in PAD patients, who therefore not only face adverse limb events but also have an increased risk of major adverse cardiovascular events (MACE), which include myocardial infarction, stroke, and cardiovascular death [5,6].

Timely recognition of individuals at risk of developing PAD and its complications would enable their early management, potentially preventing or at least slowing functional decline and major adverse events associated with the disease. Among potential predictive factors are biomarkers. Several different biomarkers were studied in patients with PAD, among them markers of immunity and inflammation, hemostasis, kidney function, extracellular matrix remodeling, metabolism, cardiac markers, transport proteins, and growth factors and hormones, but none of them has so far been accepted as a predictor of outcome in PAD patients [7,8,9,10]. This review focuses on the studies concerning the predictive role of two circulating biomarkers of tissue ischemia, high-sensitivity cardiac troponin (hs-cTn) and ischemia-modified albumin (IMA), in patients with PAD.

## 2. High-Sensitivity Troponin: A Marker of Subclinical Coronary Artery Disease in Patients with Peripheral Arterial Disease

Troponin is a regulatory protein complex formed by three subunits: troponins C, T, and I. These subunits are present in all striated muscles—skeletal and cardiac—but exist as distinct isoforms encoded by different genes. The cardiac-specific isoforms, especially cardiac troponins T and I, are released into the bloodstream when myocardial cells are damaged, making them highly sensitive and specific indicators of myocardial injury [11,12]. In the early 1990s, the first commercial assays for cardiac troponins T and I became available, allowing the introduction of troponin as a diagnostic tool in acute myocardial infarction [13]. Two decades later, assays for measuring the levels of high-sensitivity cardiac troponin T (hs-cTnT) and high-sensitivity cardiac troponin I (hs-cTnI) were developed, allowing for the detection of very low concentrations of troponin in the bloodstream [14]. This advance enabled earlier and more accurate diagnosis of myocardial infarction [15] but also led to the recognition that troponin elevations can occur in a range of non-ischemic conditions, such as heart failure, impaired renal function, sepsis, pulmonary embolism, and even strenuous exercise [16]. Furthermore, both hs-cTnT [17,18] and hs-cTnI [19] levels have been shown to predict the risk of future cardiovascular events effectively.

Following recognition of hs-cTn as a predictive biomarker, several studies have explored its clinical significance in patients with PAD. One of the earliest was a 2014 study by Pohlhammer et al. [20], who compared hs-cTnT levels in 235 male patients with IC to 249 age- and diabetes-matched controls. PAD patients were more likely to have hs-cTnT levels above the limit of detection than controls, and hs-cTnT concentrations were highly significantly related to prevalent cardiovascular disease and independently predicted MACE and all-cause mortality over a 7-year follow-up. Similar findings were reported the same year by Linnemann et al. [21] on a cohort of 1041 consecutive PAD patients undergoing endovascular peripheral artery revascularization, where detectable hs-cTnT was associated with increased one-year total mortality and amputation rates even after adjusting for age, kidney function, smoking, diabetes, and CLTI. Otaki et al. [22] similarly observed in 208 newly diagnosed PAD patients that elevated hs-cTnT independently predicted MACE and all-cause death, even after adjustment for established risk factors and biomarkers such as B-type natriuretic peptide and heart-type fatty acid-binding protein. Hikita et al. [23] explored the influence of the stage of PAD on hs-cTnT levels. They compared 42 patients with CLTI to 200 patients with IC and confirmed higher levels of hs-cTnT in CLTI patients. Furthermore, they performed coronary angiography and confirmed the presence of more severe coronary atherosclerosis in patients with CLTI. Szczeklik et al. [24] measured hs-cTnT before and after endovascular revascularization in 239 CLTI patients, showing that the post-procedural rise in hs-cTnT was associated with one-year all-cause mortality and MACE, likely reflecting clinically unrecognized coronary artery disease. Clemens et al. [25] evaluated the prognostic value of hs-cTnT in predicting mortality risk by enrolling 95 PAD patients followed for a median of 9.5 years. Even after adjusting for age, gender, diabetes, and prior cerebrovascular disease, hs-cTnT levels above the median were associated with a higher incidence of death. Cimaglia et al. [26] followed 618 diabetic patients with CLTI and foot lesions for a median of 2.7 years to assess the prognostic value of hs-cTnT. At baseline, 85% had hs-cTnT above the upper reference limit, with higher levels in those with coronary artery disease. Elevated hs-cTnT independently predicted MACE and all-cause mortality. A 2021 study by Rammos et al. [27] found that among PAD patients aged ≥80 years, hs-cTnT provided significant prognostic information for all-cause mortality in a mean follow-up time of 12.5 years, suggesting its incorporation into risk assessment models for elderly PAD patients. Of note, the results were not adjusted for atherosclerotic risk factors or comorbidities. In a 2025 study by Dobrilovic et al. [28], 487 PAD patients with advanced PAD were followed one year after endovascular revascularization to analyze the value of preprocedural hs-cTnI levels in predicting all-cause mortality or non-fatal ischemic events. While hs-cTnI above the limit of detection conferred an increased risk of the composite outcome in patients with CLTI when considering only the clinical presentation of PAD and biomarker values, its prognostic performance was lost after adjusting for clinical characteristics and common cardiovascular risk factors.

The substantial differences between studies in the study populations, follow-up duration, and defined endpoints prevent any direct comparison between studies or the drawing of consistent and generalizable conclusions regarding the prognostic value of hs-cTn in patients with PAD. An additional issue is the heterogeneity in biomarker measurement and interpretation.

Table 1 summarizes the assays for hs-cTn measurement and the reported methods for interpreting hs-cTn values in the above-mentioned studies.

Although no randomized control trials explored the predictive role of hs-cTn solely in patients with PAD, PAD patients were included in a 2017 TRA 2°P–TIMI 50 biomarker substudy by Eisen et al. [19], evaluating the prognostic value of hs-cTnT in patients with stable atherosclerotic cardiovascular disease. hs-cTnI stratified the 3-year risk of MACE in 15,833 stable patients with established atherosclerosis, among whom 17.5% had PAD. This association was predominantly driven by cardiovascular death and myocardial infarction and remained significant in symptomatic PAD and prior myocardial infarction after multivariable adjustment. Extending these findings, Marston et al. [29] demonstrated that hs-cTnI can enhance cardiovascular risk assessment in patients with a history of myocardial infarction. In a cohort of 8635 participants from the PEGASUS-TIMI 54 trial, integration of hs-cTnI levels in the atherosclerotic cardiovascular disease risk framework [30] improved risk stratification and led to the reclassification of nearly 12% of patients into more appropriate risk groups.

Several studies have also evaluated hs-cTn as a predictor of future PAD among individuals in the general population. A 2018 Atherosclerosis Risk in Communities (ARIC) study [31] included 12,288 adults free of clinical PAD at baseline. Over a median follow-up of 22 years, 454 participants (3.7%) developed PAD, including 164 cases of CLTI. The study found that elevated levels of hs-cTnT were independently associated with the future development of PAD, with the association being especially pronounced for CLTI. The authors concluded that cardiac biomarkers such as hs-cTnT may be useful in identifying individuals at particularly high risk of developing CLTI, even in the absence of clinically manifest cardiovascular disease at baseline. Results of a 2020 study by Janus et al. [32] support the role of hs-cTn as a predictor of future PAD. In their cohort of 2909 participants without known PAD or coronary artery disease from the Chronic Renal Insufficiency Cohort (CRIC) who were followed for a mean of 7.4 years, elevated hs-cTnT was a strong, independent predictor of future PAD, even after adjustment for traditional cardiovascular risk factors. Similarly, Garg et al. [33] investigated longitudinal associations between hs-cTnT and incident PAD in the Multi-Ethnic Study of Atherosclerosis (MESA). In approximately 6700 participants without baseline cardiovascular disease, higher hs-cTnT levels were independently associated with the development of clinical PAD, including cases defined by low ABI, over a median follow-up of 14 years. In a 2023 study, Hicks et al. [34] analyzed data from 4146 U.S. adults without known cardiovascular disease to assess associations between hs-cTn and lower-extremity disease. Elevated hs-cTnT and hs-cTnI were significantly associated with peripheral neuropathy but not with PAD. Among individuals with PAD, 74% had elevated hs-cTnT. Over a median follow-up of 17 years, elevated troponin levels were independently associated with increased all-cause and cardiovascular mortality, especially in participants with coexisting PAD or neuropathy. Besides the prognostic value, the authors also highlighted a high burden of subclinical cardiovascular disease, as reflected by elevated cardiac biomarkers, in persons with PAD or neuropathy.

## 3. Ischemia-Modified Albumin: A Marker of Disease Burden and Cardiovascular Complications in Patients with Peripheral Arterial Disease

IMA is a relatively recent biomarker, with research dating back approximately 30 years. In the early 1990s, researchers discovered that exposure of human serum albumin to ischemia induces its structural alterations, particularly in the N-terminal region. These alterations significantly reduce the capacity of albumin to bind transition metals, particularly cobalt, forming the biochemical basis for developing the Albumin Cobalt Binding (ACB) test for IMA detection. In 2000, Bar-Or et al. published a preliminary clinical study about the clinical usefulness of ACB as a diagnostic test for early detection of myocardial ischemia [35]. In the following years, alternative methods for IMA measuring were developed [36], and IMA was investigated in a variety of cardiovascular diseases, including acute coronary syndrome [37], stroke [38], acute mesenteric ischemia [39], PAD [40], diabetic angiopathy [41], and pulmonary embolism [42].

Studies evaluating IMA in patients with PAD are scarce and generally include small cohorts. Among the earliest is a 2004 study by Roy et al. [43], who measured IMA levels in a small cohort of 23 PAD patients with IC, exposed to an exercise-induced skeletal muscle ischemia. Authors observed that a substantial proportion of PAD patients had increased IMA concentrations despite the absence of cardiac ischemia (26% at baseline, and 48% one hour after exercise) and concluded that noncardiac ischemia could limit the usefulness of this marker for the diagnosis of myocardial ischemia. A similar conclusion has been drawn in the 2006 study by Troxler et al. [44], exploring the production of IMA during ischemia of non-cardiac tissues in a cohort of 12 patients attending for elective open transperitoneal AAA repair and 19 PAD patients with IC.

Few studies addressed the relation between IMA and the severity of PAD. In 2006, Montagnana et al. [45] published a study involving 35 PAD patients who were compared to 20 controls displaying moderate-to-high cardiovascular risk factors, but with no clinical evidence of PAD. The authors did not observe elevated IMA concentrations in PAD patients, concluding that the role of IMA as a marker of chronic peripheral ischemia remains questionable. In contrast, Gunduz et al. [46] reported higher IMA levels in 22 patients with severe limb ischemia compared to 22 healthy volunteers. Ma et al. [47] evaluated IMA in 290 patients with type 2 diabetes, of whom 110 had PAD defined as ABI < 0.90 or ≥1.30. IMA levels were significantly higher in patients with PAD and correlated positively with HbA1c. The authors suggested that IMA may serve as an additional biomarker for PAD risk stratification in type 2 diabetic patients. In 2022, Feng et al. [48] compared 110 patients with type 2 diabetes and PAD to 110 healthy controls. IMA levels were higher in the PAD group, and further stratification of the PAD cohort based on ABI index into mild, moderate, and severe subgroups revealed a positive correlation between IMA levels and PAD severity. A 2023 study by Li et al. [40] included 723 elderly participants (mean age 71 years), including healthy controls and PAD patients with either single-site or multiple-site occlusions. IMA levels were elevated in PAD patients with single-site occlusions compared to healthy controls, and even higher levels were observed in patients with multiple-site occlusions compared to those with occlusions at a single site. Ӧzsin et al. [49] evaluated IMA as a potential marker of PAD severity in 150 participants divided into three groups: 50 patients with moderate-to-severe IC, 50 with mild IC, and 50 healthy volunteers. IMA levels were significantly higher in PAD patients compared to healthy individuals, with the highest levels observed in those with moderate-to-severe claudication.

In 2007, Hacker et al. [50] published a study exploring the effect of lower limb arteries revascularization on IMA in 21 PAD patients with IC. Baseline IMA levels of PAD patients were higher compared to the control group of 96 participants. Furthermore, they increased 30 min after the intervention, suggesting a correlation between IMA concentration and plaque disruption.

In 2015, Falkensammer et al. [51] evaluated the prognostic value of IMA in 66 male patients with IC who were followed for a mean period of 3.6 years. They found that elevated IMA levels were a strong independent predictor of MACE and overall mortality. In a 2025 study by Dobrilovic et al. [28], including PAD patients after lower limb endovascular revascularization, detectable levels of IMA were an independent predictor of ischemic events and all-cause death in a subgroup of 237 patients with CLTI, but not in the whole cohort, which also included participants with IC.

In 2018, Nativel et al. [52] investigated the prognostic value of IMA for the development of PAD in individuals with type 2 diabetes. A total of 1412 participants were followed over a median period of 5.6 years, during which 7.9% developed advanced PAD requiring lower extremity revascularization or amputation. The study found that elevated IMA levels were independently associated with an increased risk of PAD. However, IMA did not provide incremental predictive value beyond traditional cardiovascular risk factors for PAD development.

Table 2 summarizes the assays for IMA measurement and the reported methods for interpreting IMA values in the above-mentioned studies.

## 4. Discussion

In recent years, research interest in prognostic biomarkers has increased in the context of personalized medicine and the emerging possibilities offered by artificial intelligence. AI could facilitate the development of complex diagnostic and predictive algorithms, enhancing risk stratification, guiding individualized management in patients with PAD, and improving the identification of individuals at higher risk of developing PAD [53,54]. Among the most obvious targets of interest in circulation are inflammatory biomarkers, as atherosclerosis is fundamentally an endothelial inflammatory disease [55], and anti-inflammatory therapies have been shown to reduce cardiovascular events [56]. Prothrombotic biomarkers are also of particular interest, since thrombus formation plays a central role in ischemic events, and markers such as fibrinogen and D-dimer have been associated with increased future cardiovascular risk [57,58]. Finally, biomarkers of cardiac ischemia reflect the generalized, polyvascular nature of atherosclerosis. Defined as the simultaneous presence of clinically relevant obstructive atherosclerotic lesions in at least two major arterial territories, polyvascular disease independently increases risk of MACE, with event rates rising with the number of affected arterial beds [5]. Among patients with cardiovascular disease, those with PAD are most likely to present with polyvascular disease, and they most frequently die from myocardial infarction and stroke rather than limb-related complications [59].

Hs-cTn, as a marker of subclinical myocardial injury, holds strong potential as a prognostic predictor in patients with PAD. Higher levels of hs-cTn in PAD patients in comparison to controls could point to the presence of polyvascular disease with concurrent involvement of coronary arteries. Indeed, studies have linked hs-cTn concentrations with atherosclerotic changes observed on coronary angiography [23] and confirmed its connection with the adverse outcome in stable patients with previous myocardial infarction [19]. Interestingly, elevated levels of hs-cTn in individuals from the general population have been independently associated with the future development of PAD [31,33], possibly reflecting the chronic, progressive nature of atherosclerosis which begins long before the symptoms become apparent.

In contrast, less is known about IMA. First, it has not been studied as extensively as hs-cTn; there is no accepted threshold for pathological levels of IMA, and consequently, most studies do not use a cut-off value but rather analyze IMA as a continuous variable. And second, IMA levels reflect oxidative stress, acidosis, and free radical activity, and can therefore also be elevated in the absence of ischemia [36,60].

In the context of an aging population and increasingly sedentary lifestyle, PAD represents a growing global health problem, significantly impairing patients’ quality of life and imposing a considerable financial burden on healthcare systems. However, not all patients with PAD face the same risk of disease progression or adverse cardiovascular outcomes. The reported 5-year cumulative incidence of progression was 7% from asymptomatic PAD to IC, and 21% from IC to CLTI [61]. Prognostic stratification currently relies primarily on the clinical presentation of PAD. Compared with patients with an ABI < 0.9, those with CLTI exhibited a more than 2-fold higher risk of all-cause mortality and myocardial infarction, and almost 4-fold higher risk of major amputation [6]. Available evidence consistently shows that hs-cTn levels not only differentiate patients with PAD from those without, but also parallel disease severity within the PAD spectrum. Patients with PAD generally have higher troponin levels than controls, and among PAD patients, those with CLTI exhibit significantly higher concentrations compared to those with IC. This gradient suggests that the more advanced the clinical presentation of PAD, the greater the degree of subclinical myocardial injury or concomitant cardiovascular burden, reflected by rising hs-cTn. In line with this, higher troponin levels in CLTI patients are linked with more severe coronary atherosclerosis and translate into worse outcomes, including increased mortality, amputation, and MACE. Thus, hs-cTn could serve not only as a prognostic biomarker but also as a surrogate of overall cardiovascular risk that escalates in parallel with PAD severity. Less is known about IMA, but existing evidence suggests that its levels in PAD follow a severity-dependent gradient, with higher concentrations in patients with severe ischemia, CLTI, or multilevel occlusions. Interestingly, Dobrilovic et al. demonstrated that CLTI patients with undetectable IMA had a prognosis comparable to those with IC [28]. This finding raises the possibility that clinical assessment—often subjective—may be less reliable than biomarker-based evaluation, or that biomarkers such as IMA carry greater prognostic value than clinical presentation alone. Further research is needed to clarify this relationship.

Although both hs-cTn and IMA have been associated with the severity and prognosis of PAD, their current clinical applicability for risk stratification remains limited. The present body of evidence has restricted usability due to substantial heterogeneity in study populations, biomarker measurement methods, follow-up duration, and defined endpoints. In addition, the interpretation of biomarker prognostic value in PAD is further complicated by the presence of multiple confounding factors. Circulating biomarker levels, as well as the disease itself, may be influenced by age, sex, renal function, and presence of systemic inflammation, all of which are independently associated with adverse cardiovascular outcomes. The coexistence of major atherosclerotic risk factors such as diabetes, smoking, hypertension, and dyslipidemia, as well as the presence of coronary or cerebrovascular disease, can obscure the independent contribution of a single biomarker to prognosis. Moreover, the use of pharmacological therapies, including statins, antiplatelet agents, and anti-inflammatory drugs, may modify biomarker expression or downstream pathways, further confounding the observed associations. In this review, we focused on two representative biomarkers investigated as predictors of outcome in PAD patients to illustrate the major methodological limitations observed across current studies. In our opinion, two key challenges for future research can be identified. First, there is a lack of randomized controlled trials specifically focusing on patients with PAD. Second, standardized prognostic cut-off values are lacking. Heterogeneous thresholds used across studies to define biomarker positivity have resulted in inconsistent outcome reporting and, consequently, limited comparability between studies. A consensus on standardized cut-off values would facilitate their broader adoption in risk stratification and management of PAD patients. For example, the 2022 ESC Guidelines on cardiovascular assessment and management of patients undergoing non-cardiac surgery recommend measuring hs-cTnT or hs-cTnI in individuals with known cardiovascular disease, cardiovascular risk factors (including age ≥ 65 years), or symptoms suggestive of cardiovascular disease. However, rather than specifying absolute cut-off values, the guidelines advise monitoring perioperative changes in hs-cTn concentrations [62].

To conclude, hs-cTn and IMA are two of the many studied biomarkers that show promising potential as predictors of outcomes in patients with PAD. However, before implementation of any of the biomarkers into the clinical guidelines or risk score calculators, their optimal cut-off values should be established, and their usefulness confirmed and weighed in randomized trials. The currently known strengths and limitations of each biomarker are summarized in Table 3.

## Figures and Tables

**Table 1:** Assays for measuring the high-sensitivity cardiac troponin (hs-cTn) and the type of analysis reported in studies investigating the clinical significance of hs-cTn in patients with lower extremity peripheral arterial disease.

Study (1st Author, Year)	Assay (as Reported)	Analysis of hs-cTn
Pohlhammer, 2014 [20]	hs-cTnT, 5th gen. Roche E170 platform	Categorical; <LoD (5 ng/L) defined as LoD/√2; ≥5 ng/L vs. <5 ng/L; and ≥14 ng/L (99th percentile) vs. <14 ng/L.
Linnemann, 2014 [21]	cTnT, 4th gen. Roche Elecsys (Cobas 6000 e601)	Categorical; cut-off = 0.01 ng/mL (99th percentile); ≥0.01 ng/mL vs. <0.01 ng/mL.
Otaki, 2015 [22]	hs-cTnT, 4th gen. Roche Elecsys 2010	Continuous (log_10_ hsTnT) and tertiles (<0.90 ng/mL, 0.90–1.23 ng/mL, and >1.23 ng/mL); higher tertiles predicted MACE.
Hikita, 2015 [23]	hs-cTnT, Roche Elecsys	Continuous; normal range < 0.014 ng/mL; hs-cTnT higher in patients with CLTI vs. IC.
Szczeklik, 2018 [24]	hs-cTnT, Roche Elecsys 2010	Post-procedural hs-cTnT ≥ 14 ng/L plus ≥ 30% relative increase from baseline defined as a MI.
Clemens, 2019 [25]	hs-cTnT, Roche Cobas e601	Continuous; cut-off = 9 ng/L for predicting mortality in patients with PAD.
Cimaglia, 2021 [26]	hs-cTnT Roche Elecsys Cobas e601	Continuous; cut-off = 25 ng/L for predicting MACE in patients with diabetes and CLTI (without known CAD).
Rammos, 2021 [27]	Troponin-ultra	Categorical; below vs. above 99th percentile (>40 ng/L vs. ≤40 ng/L).
Dobrilovic, 2025 [28]	hs-cTnI, Abbott Alinity I	Categorical; detectable vs. undetectable (>10 ng/L vs. <10 ng/L).

LoD = level of detection; CLTI = chronic limb-threatening ischemia; IC = intermittent claudication. MI = myocardial injury; PAD = peripheral arterial disease; MACE = major adverse cardiovascular event; CAD = coronary artery disease.

**Table 2:** Assays for measuring the ischemia-modified albumin (IMA) and the type of analysis reported in studies investigating the clinical significance of IMA in patients with lower extremity peripheral arterial disease (PAD).

Study (1st Author, Year)	Assay (as Reported)	Analysis of IMA Values
Roy, 2004 [43]	ACB, Cobas MIRA PLUS	Continuous; baseline and serial peri-exercise IMA values inversely correlated with ABI. No fixed cut-off.
Troxler, 2006 [44]	ACB, Cobas MIRA PLUS	Continuous; preoperative IMA higher in patients with IC vs. AAA and controls, and increased with clamping in IC and AAA vs. controls; mild perioperative increase in controls. No fixed cut-off.
Montagnana, 2006 [45]	ACB, Modular System P	Continuous; no difference in patients with IC vs. control. No fixed cut-off.
Hacker, 2007 [50]	ACB, Modular System P	Continuous; IMA elevated at baseline and increased transiently after PVI in patients with IC. No fixed cut-off.
Gunduz, 2008 [46]	ACB	Results reported as absorbance units; IMA higher in patients with severe limb ischemia vs. controls. Cut-off = 0.22 absorbance units.
Ma, 2011 [47]	ACB, Cobas MIRA PLUS	Continuous; IMA higher in patients with PAD vs. those without PAD. No fixed cut-off.
Falkensammer, 2015 [51]	ACB, Modular System P	Continuous; IMA higher in IC patients who developed MACE. Prognostic cut-off >103.9 kU/L.
Nativel, 2018 [52]	ACB	Continuous and tertiles; high IMA associated with increased risk of major PAD in patients with diabetes. No fixed cut-off.
Feng, 2022 [48]	ACB	Continuous; IMA correlated with PAD severity in diabetic patients. No fixed cut-off.
Li, 2023 [40]	ELISA	Results reported as absorbance units; IMA increased with number of artery occlusions. No fixed cut-off
Özsin, 2024 [49]	ACB	Continuous; IMA higher in patients with IC vs. controls and correlated with disease severity. Cut-off = 0.802 U/mL predicted presence of PAD.
Dobrilovic, 2025 [28]	ELISA	Categorical; detectable vs. undetectable based on LoD (0.48 U/L).

ACB = albumin cobalt binding; ABI = ankle brachial index; IC = intermittent claudication; AAA = abdominal aortic aneurysm; PVI = peripheral vascular intervention; MACE = major adverse cardiovascular event; LoD = level of detection.

**Table 3:** Strengths and limitations of high-sensitivity cardiac troponin and ischemia-modified albumin as predictors in patients with lower extremity peripheral arterial disease (PAD).

**Cardiac troponin** 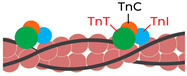
**Strengths**
Highly sensitive and specific for myocardial injury.
Correlates with PAD severity; higher levels are seen in advanced PAD (CLTI vs. IC).
Predictive value for MACE, mortality, and amputation in PAD patients.
Reflects subclinical myocardial injury and the presence of PVD in PAD patients.
Predicts future PAD development even in asymptomatic individuals.
Widely available and standardized assays.
Part of guideline-supported cardiovascular risk stratification.
**Limitations**
Heterogeneous cut-off values across studies limit the comparability of results.
Lack of randomized controlled trials specifically in PAD populations.
Non-specific elevation in renal failure, sepsis, heart failure, etc.
Unclear incremental predictive value beyond clinical characteristics and risk factors.
**Ischemia-modified albumin** 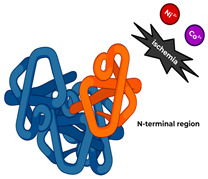
**Strengths**
Sensitive to ischemia, including skeletal muscle ischemia.
Reflects oxidative stress, acidosis, and free radical activity linked to ischemia.
Levels increase with PAD severity, especially in CLTI.
May predict ischemic events and all-cause mortality in PAD patients with CLTI.
May offer incremental prognostic information beyond clinical assessment.
**Limitations**
Less extensively studied than troponin; small, heterogeneous cohorts.
Heterogeneous assays and cut-off values across studies limit comparability of results.
Unclear incremental predictive value beyond traditional risk factors.
Non-specific elevation in non-ischemic oxidative stress conditions.

CLTI = chronic limb-threatening ischemia; IC = intermittent claudication; MACE = major adverse cardiovascular event; PVD = polyvascular disease.

## Data Availability

No new data were created or analyzed in this study. Data sharing does not apply to this article.

## Abbreviations

The following abbreviations are used in this manuscript:

PAD	Peripheral Arterial Disease
hs-cTn	High-Sensitivity Cardiac Troponins
IMA	Ischemia-Modified Albumin
ABI	Ankle-Brachial Index
IC	Intermittent Claudication
CLTI	Chronic Limb-Threatening Ischemia
MACE	Major Adverse Cardiovascular Event
hs-cTnT	High-Sensitivity Cardiac Troponin T
hs-cTnI	High-Sensitivity Cardiac Troponin I
